# Based on knowledge capital value for disease cost accounting of diagnosis related groups

**DOI:** 10.3389/fpubh.2024.1269704

**Published:** 2024-06-10

**Authors:** Jinli Duan, Feng Jiao, Jicheng Xi, Qichun Zhang

**Affiliations:** ^1^School of Economics and Management, Sanming University, Sanming, Fujian, China; ^2^School of Leadership and Managment, Arden University, Coventry, United Kingdom; ^3^School of Economics and Management, Fuzhou University, Fuzhou, China; ^4^School of Economics and Management, Yango University, Fuzhou, Fujian, China

**Keywords:** knowledge capital value, cost accounting, diagnosis related groups, medical workers, analytic hierarchy process

## Abstract

**Background:**

The National Health Commission and the other relevant departments in China have initiated testing of the Diagnosis Related Groups (DRGs) system in 30 pilot locations since 2019. In the process of DRG payment reform, accounting for the costs of diseases has become a highly challenging issue. The traditional method of disease accounting method overlooks the compensation for the knowledge capital value of medical personnel.

**Objective:**

The primary objective of this study is to analyze the cost accounting scheme of China’s Diagnosis Related Groups (C-DRG), focusing on the value of knowledge capital.

**Methods:**

The study initially proposes a measurement index system for the value of knowledge-based capital, including the difficulty of disease treatment, labor intensity of disease treatment, risk of disease treatment, and operation/treatment time for diseases. The Analytic Hierarchy Process (AHP) is then utilized to weigh the features of medical workers’ knowledge capital value. First, pairwise comparisons are conducted in this stage to develop a two-pair judgment matrix of the primary indicators. Second, the eigenvectors corresponding to the maximum eigenvalues of the matrix are calculated to generate the weight coefficient of each feature. The consistency test is carried out after this stage. An empirical analysis is conducted by collecting data, including the full costs of treating three types of diseases—hip replacement, acute simple appendicitis, and heart bypass surgery—from one public medical institution.

**Results:**

The empirical analysis examines whether this DRG costing accounting can address the issue of neglecting the value of medical workers’ knowledge capital. The methods reconfigure the positive incentive mechanism, stimulate the endogenous motivation of the medical service system, foster independent changes in medical behavior, and achieve the goals of reasonable cost control.

**Conclusion:**

In the cost accounting system of C-DRG, the value of medical workers’ knowledge capital is acknowledged. This acknowledgment not only boosts the enthusiasm and creativity of medical workers in optimizing and standardizing the diagnosis and treatment process but also improves the transparency and authenticity of DRG pricing. This is particularly evident in the optimization and standardization of the diagnosis and treatment processes within medical institutions and in monitoring inadequate medical practices within these institutions.

## Introduction

Knowledge capital encompasses both explicit and implicit knowledge owned or controlled by an organization, capable of bringing value to it (1). This concept aligns with intangible assets (knowledge capitals) in traditional accounting practices (2) and aids in discerning disparities between an organization’s book value and market value. However, recent research has criticized practitioners and academics for being ensnared by the notion of ‘Quantification’, assuming that because some knowledge capital cannot be objectively and fairly measured, it should not be included in the cost element (3).

Furthermore, the measurement and disclosure of knowledge capital in real practice primarily concentrate on the static knowledge capital stock, neglecting the dynamic aspect of knowledge capital in ‘action’ (4). In this study, we propose a disease cost accounting method based on the value of knowledge capital within the DRG payment system.

Knowledge-based human capital constitutes the fundamental production factor in medical institutions. However, due to the scarcity of knowledge capital and ownership separation of knowledge capital, as well as the high acquisition costs associated with it, the cost accounting method for knowledge capital differs from the traditional costing accounting method (5). In addition, the organizational structure and service process management in medical institutions diverge significantly from those in traditional manufacturing industries (6). Consequently, specialized cost accounting and management models are necessary for medical institutions (7). The cost accounting system based on manufacturing costs is inadequate for the accounting system in medical institutions, as it cannot reflect the intrinsic logic of innovative behaviors of medical workers and the core value of innovative elements in medical institutions (8).

As integrated innovators, medical workers, particularly doctors who work on the frontlines, contribute valuable implicit knowledge accumulated from their long-term service (9). This knowledge enables them to manage uncertainties for patients and provide value-added services for medical institutions. According to Becker’s related theories, the time required to accomplish a task comprises two components: the time required to perform the task and the time needed to acquire the knowledge necessary to complete the task (10). Therefore, the time necessary for medical workers to execute the integration and coordination of medical services should encompass two aspects, including the time allocated for delivering basic services and the time dedicated to integrating and coordinating medical services. The former entails providing medical services to patients, such as the time spent on consultation, decision-making, and evaluating decisions around diagnostic and treatment activities. The latter involves accumulating activities essential for effectively innovating medical activities. In reality, the process of acquiring knowledge often requires more time than making and evaluating decisions.

Significant quantities of implicit knowledge demand extended periods for accumulation (11–15). This implicit knowledge requisite for medical integration activities encompasses disease-specific insights, patient-specific knowledge, medical diagnostic and treatment technologies, including drug treatment methodologies, and insights into the behavioral habits and foundational knowledge of relevant medical team members, essential for effectively navigating uncertainty (14, 16–19). This part of knowledge necessitates substantial accumulation in the initial education stage; meanwhile, it highly relies on the accumulation of doctors’ long-term clinical practice. Furthermore, doctors must consistently attend to patients to effectively apply relevant expertise. In this regard, current costing processes applied in medical institutions struggle to acknowledge the working time that medical workers invest in the abovementioned service, particularly the time required for medical workers to accumulate and complete tasks. The corresponding value of this time is rarely acknowledged (20, 21).

Full cost accounting of diseases considers the value of knowledge capital essential for medical workers to carry out medical service projects. The value of medical workers’ knowledge capital primarily manifests in the realization pathway, encompassing explicit and tacit knowledge (22–24). Explicit knowledge capital primarily pertains to the professional qualifications and other attributes of medical personnel, while tacit knowledge primarily pertains to their risk perception and ability to navigate uncertainty in medical service projects (25–28). Tacit knowledge is mainly evaluated through the difficulty and risk coefficients associated with medical services performed by medical workers for specific diseases (29, 30).

The rest of this article is organized as follows: Section 2 examines the evaluation of medical workers’ knowledge capital within the DRG cost accounting system. Section 3 provides an empirical analysis illustrating the cost accounting process based on knowledge of capital value. Section 4 applies the discussion to the cost accounting result. Finally, Section 5 offers a conclusion and proposes potential future research avenues.

## Methods

### The full-cost accounting index system

The disease cost index system includes labor costs, drug costs, health material costs, inspection costs, and depreciation and sharing costs (31). The calculation of labor costs is based on the evaluation model of the value of the knowledge capital of medical workers (32), including 4 primary indicators, namely the difficulty of the disease project, the labor intensity of the disease project, the risk degree of the disease project, and the operation time of the disease project, and 11 secondary indicators, which are the difficulty of disease treatment, level of technical commitment, knowledge requirement for operators, the requirement for operator’s decision - making ability, levels of physical exertion per unit of time, ability, levels of physical exertion per unit of time, levels of concentration per unit time, risk hazards for patients in treatment, occupational exposure of medical workers, consultation time (including surgery time), time for nursing and time for examining. These indicators are summarized and shown in Table 1.

**Table 1:** Disease cost accounting index system.

Main costs	Primary Indicator	Secondary Indicator
Labor costs	Difficulty of disease treatment (D)	The difficulty of disease treatment (D1)
		Level of technical commitment (D2)
		Knowledge requirement for operators (D3)
		The requirement for operators’ decision-making ability (D4)
	Labor intensity of disease treatment (I)	Levels of physical exertion per unit of time(I1)
		Levels of concentration per unit of time(I2)
	Risk of disease treatment (R)	Risk hazards for patients in treatment (R1)
		Occupational exposure of medical workers (R2)
	Operations/Treatment time of diseases (T)	Consultation time (including surgery time) (T1)
		Time for nursing(T2)
		Time for examing(T3)
Costs of Medicines	Western medicines	
	Proprietary Chinese medicines	
	Chinese herbal medicines	
Costs of sanitary materials	Fees for radioactive materials	
	Materials for blood transfusion	
	Medical used oxygen	
	Laboratory materials	
	Other hygiene materials	
Examine costs	Laboratory tests	
	Radiological examing	
Allocation cost of Depreciation	Depreciation of fixed assets	
	Amortization of intangible assets	
	Amortization of office utilities and internet bills	

### Determination of weight

In this study, the analytic hierarchy process (AHP) was used to determine the weight of medical workers’ knowledge of cost accounting indicators.

#### The establishment of a hierarchical framework

At the highest level of the hierarchy usually lies a single element, which is the decision goal. The intermediate level comprises criteria and sub-criteria, which may further branch into multiple layers. The criteria are guided by the decision-making goal, and the sub-criteria are influenced by the criteria at the preceding level, reflecting a top-down dominance relationship within the hierarchy. The knowledge capital value of costing indicators for medical workers is shown in Table 2.

**Table 2:** Knowledge capital value costing indicators for medical workers.

The difficulty of disease treatment	Labor intensity of disease treatment	Risks of disease treatment	The operating time of disease treatment
D_1_: The difficulty of disease treatment	I_1_: Levels of physical exertion per unit of time	R_1_: Risk hazards for patients in treatment	T_1_: Consultation time (including surgery time)
D_2_: Level of technical commitment	I_2_:Levels of concentration per unit of time	R_2_: Occupational exposure of medical workers	T_2_: Time for nursing
D_3_: Knowledge requirement for operators			T_3_: Time for examing
D_4_: The requirement for operators’ decision-making ability			

#### The construction of a two-pair judgment matrix

Once the hierarchical framework is established, the connection between the upper and lower elements becomes apparent. The expert group conducts an in-depth analysis of the accounting book data. Simultaneously, they invite cost accountants, clinicians, nurses, and medical technicians from the public hospitals where DRG trials are taking place. These participants are tasked with comparing the importance of indicators and constructing a two-by-two comparison matrix. The analytic hierarchy method typically employs the 9-level scale method to assign values to the elements of the judgment matrix, as shown in Table 3.

**Table 3:** Scale explanation on value 1 to 9.

Value	Importance	The importance level in Two-by-two comparison
1	Equally important	The i element is just as important as the j element
3	Slightly stronger	The i element is slightly more important than the j element
5	strong	The i element is obviously more important than the j element
7	Very strong	The i element is more important than the j element
9	Absolutely strong	The i element is absolutely more important than the j element
2, 4, 6, 8		Scale values corresponding to intermediate states between two judgments
Reciprocal		If the j element is compared with the i element, the judgment value is the reciprocal of the aforementioned scale value

Table 3 shows that a value of 9 means “absolutely important,” a value of 7 means “very important,” and a value of 5 means “important,” so a median rating such as “the degree of importance is between very important and important” is awarded 6 points. The experts evaluated and weighed four primary indicators of the cost of knowledge for medical workers, and the resulting pairwise matrix is scored as shown in Table 4.

**Table 4:** Pairwise comparison of expert scores on decision criteria.

Compare in pairs	A more important criterion	Numerical level
D-I	D	7
D-R	R	5
D-T	D	6
I-R	R	5
I-T	I	1
R-T	R	7

According to the scoring table provided by the decision expert, a pairwise comparison judgment matrix A of the primary indicators can be constructed:


$$A=\left(\begin{array}{cccc} 1 & 7 & 5 & 6\\ \frac{1}{7} & 1 & 5 & 1\\ \frac{1}{5} & \frac{1}{5} & 1 & 7\\ \frac{1}{6} & 1 & \frac{1}{7} & 1 \end{array}\right)$$

#### The calculation of the weight coefficient

In brief, the eigenvectors corresponding to the maximum eigenvalues of matrix A can be calculated by approximate calculation methods such as the square root method $\lambda_{\text{max}}$ and normalized to the weight of each evaluation index. The calculation of the weight coefficient is divided into three steps, using the square root method. The specific steps to adopt the square root method are as follows:

(1) calculate the product $M_{i}$ of all elements $a_{ij}$ of each row of the judgment matrix A.


$$M_{i}=\overset{j=1}{\underset{n}{\Pi }}a_{ij}$$

$$M_{1}=210$$

$$M_{2}=0.7143$$

$$M_{3}=7$$

$$M_{4}=0.0238$$

(2) calculate the $n^{th}$ root $\beta_{i}$ of $M_{i}$


$$\beta_{i}=\sqrt[{n}]{M_{i}}$$

$$\beta_{1}=3.8068$$

$$\beta_{2}=0.9193$$

β=31.6266

$$\beta_{4}=0.3928$$

(3) Normalization of vectors $\beta ={\left(\beta_{1},\beta_{2},\ldots ,\beta_{n}\right)}^{T}$


$$\omega_{j}=\frac{\beta_{j}}{\overset{n}{\underset{j=1}{\sum }}\beta_{j}},\left(j=1,2,\ldots ,n\right)$$

Then, the vector $\omega =\left(\omega_{1},\omega_{2},\ldots \omega_{n}\right)$ is the desired feature vector, and the weight of the primary index is obtained $\omega =\left(0.4643,0.1564,0.2411,0.1382\right)$ .

#### Consistency test

Upon obtaining the judgment matrix of the primary indicators, a consistency test is conducted. Due to the considerable number of pairwise comparisons, achieving complete consistency can be challenging. In reality, some inconsistency is inevitable in any pairwise comparison. To mitigate this issue, AHP offers a method to gauge the consistency of decision-makers when making such comparisons. If the desired level of agreement is not met, decision-makers should reassess the pairwise comparisons and make necessary adjustments before proceeding with the AHP analysis to minimize bias in subjective judgment. The consistency of pairwise comparisons is measured through a consistency metric (Table 5). The consistency check is conducted in three steps:

**Table 5:** The corresponding R.I value.

Matrix order	1	2	3	4	5	6	7	8	9
R.I	0	0	0.52	0.90	1.12	1.26	1.36	1.41	1.46

The first step is to calculate the maximum eigenroot $\lambda_{\text{max}}$ in the pairwise comparison matrix *A*


$$\lambda_{\text{max}}=\overset{n}{\underset{i=1}{\sum }}\frac{{\left(A\omega \right)}_{i}}{n\omega_{i}}$$

Then,


$$A\omega =\left(\begin{array}{cccc} 1 & 7 & 5 & 6\\ \frac{1}{7} & 1 & 5 & 1\\ \frac{1}{5} & \frac{1}{5} & 1 & 7\\ \frac{1}{6} & 1 & \frac{1}{7} & 1 \end{array}\right)\left(0.4643,0.1564,0.2411,0.1382\right)$$

which calculated $\lambda_{\text{max}}=4.035$ .

Step 2: Calculate consistency indicator C.I


$$C.I=\frac{\lambda_{\text{max}}-n}{n-1}=0.0117<0.1$$

Step 3: Calculate the consistency ratio C.R


$$C.R=\frac{C.I}{R.I}=\frac{0.0117}{0.90}=0.013<0.1$$

Since C.I<0.1 and C.R<0.1 , it is acceptable to compare the inconsistencies of matrix *A*, that is, the obtained weight–weight coefficients are valid. Similarly, the weights of secondary indicators can be obtained, and the summary table of the weights of primary and secondary indicators is shown in Table 6.

**Table 6:** Weight of knowledge capital value of accounting indicators for medical workers.

**Indicator**	**D**	**I**	**R**	**T**
	0.4643	0.1564	0.2411	0.1382
The difficulty of disease treatment	0.2015	/	/	/
	0.1107			
	0.0572			
	0.0949			
Labor intensity of disease treatment	/	0.0965	/	/
		0.0599		
Risks of disease treatment	/	/	0.1607	/
			0.0804	
The operation time of disease treatment	/	/	/	0.0861
				0.0184
				0.0237

### Empirical analysis

This study collects data on the full costs of treating three types of diseases: hip replacement, acute simple appendicitis, and heart bypass surgery. The dataset includes a patient settlement list, the first page of medical records, and the knowledge costs of medical workers, all sourced from a single public hospital. Specifically, the components of full cost within DRG encompass the knowledge capital of medical workers, sanitary material costs (blood transfusion cost, oxygen usage cost, costs of imaging materials, and costs of laboratory materials), drug costs, depreciation expense of fixed assets, amortization expense of intangible assets, withdrawal of medical risk fund, and other operating expenses (office expenses, utility costs, costs of postage and telecommunications, internet bills, official car operation and maintenance fees and travel expenses, training fees, union funds, and other expenses) (33).

The depreciation expenses of fixed assets and office expenses, salaries of management staff, and utilities and Internet bills that need to be apportioned in disease treatment are shown in Table 7.

**Table 7:** Calculation table of disease sharing costs.

Apportionment level	Apportionment parameters	Apportioned items	Formula
Cost aggregation	Apportioned according to the proportion of medical expenses	Utilities cost	The cost of utilities and other expenses share in the disease treatment = $\mathrm{The}\;\mathrm{cost}\;\mathrm{of}\;\mathrm{utilities}\;\mathrm{borne}\;by\;\mathrm{the}\;\mathrm{department}\times \frac{\mathrm{The}\;\mathrm{cost}\;\mathrm{of}\;\mathrm{disease}\;\mathrm{treatement}}{\mathrm{The}\;\mathrm{overall}\;\mathrm{medical}\;\mathrm{expenses}\;\mathrm{of}\;\mathrm{the}\;\mathrm{department}\;}$
First-level apportionment	apportioned according to the proportion of medical workers in disease treatment	Administrative and logistics management costs	The cost of administrative and logistics management = $\frac{\mathrm{The}\;\mathrm{number}\;\mathrm{of}\;\mathrm{staff}\;\mathrm{in}\;\mathrm{the}\;\mathrm{department}}{\mathrm{The}\;\mathrm{overall}\;\mathrm{staff}\;\mathrm{in}\;\mathrm{the}\;\mathrm{hospital}}\times \mathrm{The cost of adminstrative and logistics management}\times \frac{\mathrm{The}\;\mathrm{cost}\;\mathrm{of}\;\mathrm{disease}\;\mathrm{treatement}}{\mathrm{The}\;\mathrm{overall}\;\mathrm{medical}\;\mathrm{expenses}\;\mathrm{of}\;\mathrm{the}\;\mathrm{department}\;}$
Secondary allocation	Apportioned according to the income from disease treatment	Registration fee	The registration cost for disease treatment = $\frac{\mathrm{The}\;\mathrm{income}\;\mathrm{of}\;\mathrm{the}\;\mathrm{department}}{\mathrm{The}\;\mathrm{overall}\;\mathrm{income}\;\mathrm{of}\;\mathrm{the}\;\mathrm{hospital}}\times \mathrm{The current registation cost}\times \frac{\mathrm{The}\;\mathrm{cost}\;\mathrm{of}\;\mathrm{disease}\;\mathrm{treatement}}{\mathrm{The}\;\mathrm{overall}\;\mathrm{medical}\;\mathrm{expenses}\;\mathrm{of}\;\mathrm{the}\;\mathrm{department}\;}$
	Apportioned according to workload	Outpatient office costs	Outpatient office costs in disease treatment = $\frac{\mathrm{The}\;\mathrm{number}\;\mathrm{of}\;\mathrm{patients}\;\mathrm{in}\;\mathrm{the}\;\mathrm{department}}{\mathrm{The}\;\mathrm{overall}\;\mathrm{patients}\;\mathrm{in}\;\mathrm{the}\;\mathrm{hospital}}\times \mathrm{The current cost of outpatient office cost}\times \frac{\mathrm{The}\;\mathrm{number}\;\mathrm{of}\;\mathrm{patients}\;\mathrm{in}\;\mathrm{this}\;\mathrm{disease}\;\mathrm{treatement}}{\mathrm{The}\;\mathrm{overall}\;\mathrm{patients}\;\mathrm{in}\;\mathrm{the}\;\mathrm{department}\;}$
		Inpatient and food supply	Costs of inpatient and food supply in disease treatment = $\frac{\mathrm{The}\;\mathrm{number}\;\mathrm{ofinpatients}\;\mathrm{in}\;\mathrm{the}\;\mathrm{department}}{\mathrm{The}\;\mathrm{overall}\;\mathrm{inpatients}\;\mathrm{in}\;\mathrm{the}\;\mathrm{hospital}}\times \mathrm{The current cost of inpatient department}\times \frac{\mathrm{The}\;\mathrm{number}\;\mathrm{of}\;\mathrm{inpatients}\;\mathrm{in}\;\mathrm{this}\;\mathrm{disease}\;\mathrm{treatement}}{\mathrm{The}\;\mathrm{overall}\;\mathrm{inpatients}\;\mathrm{in}\;\mathrm{the}\;\mathrm{department}\;}$
	Apportioned according to the total value of the proprietary equipment	Department of Health Instruments	Costs of health instruments= $\begin{array}{l} \frac{\mathrm{The}\;\mathrm{value}\;\mathrm{of}\;\mathrm{fixed}\;\mathrm{proprietary}\;\mathrm{equipment}\;\mathrm{in}\;\mathrm{the}\;\mathrm{department}}{\mathrm{The}\;\mathrm{overall}\;\mathrm{value}\;\mathrm{of}\;\mathrm{fixed}\;\mathrm{proprietary}\;\mathrm{equipment}\;\mathrm{in}\;\mathrm{the}\;\mathrm{hospital}}\times \mathrm{The current cost of deparment of health instruments}\\ \times \frac{\mathrm{The}\;\mathrm{value}\;\mathrm{of}\;\mathrm{fixed}\;\mathrm{proprietary}\;\mathrm{equipment}\;\mathrm{in}\;\mathrm{this}\;\mathrm{disease}\;\mathrm{treatement}}{\mathrm{The}\;\mathrm{overall}\;\mathrm{value}\;\mathrm{of}\;\mathrm{fixed}\;\mathrm{proprietary}\;\mathrm{equipment}\;\mathrm{in}\;\mathrm{the}\;\mathrm{department}\;} \end{array}$
	Apportioned according to the number of discharged patients	Department of Medical History	Costs of the Department of Medical History $\begin{array}{l} \frac{\mathrm{The}\;\mathrm{number}\;\mathrm{of}\;\mathrm{discharged}\;\mathrm{patients}\;\mathrm{in}\;\mathrm{the}\;\mathrm{department}}{\mathrm{The}\;\mathrm{number}\;\mathrm{of}\;\mathrm{discharged}\;\mathrm{patients}\;\mathrm{in}\;\mathrm{the}\;\mathrm{hospital}}\times \mathrm{The current cost of Deparment of Medical History}\\ \times \frac{\mathrm{The}\;\mathrm{number}\;\mathrm{of}\;\mathrm{discharged}\;\mathrm{patients}\;\mathrm{in}\;\mathrm{this}\;\mathrm{disease}\;\mathrm{treatement}}{\mathrm{The}\;\mathrm{number}\;\mathrm{of}\;\mathrm{discharged}\;\mathrm{patients}\;\mathrm{in}\;\mathrm{the}\;\mathrm{department}\;} \end{array}$
	Apportioned according to patients in a type of diseases	Departments of medical supply, oxygen supply, and other medical assistance	Costs of departments of medical supply, oxygen supply, and other medical assistance = $\begin{array}{l} \frac{\mathrm{The}\;\mathrm{service}\;\mathrm{consumption}\;\mathrm{in}\;\mathrm{one}\;\mathrm{assistantive}\;\mathrm{department}}{\mathrm{The}\;\mathrm{service}\;\mathrm{capacity}\;\mathrm{in}\;\mathrm{one}\;\mathrm{assistantive}\;\mathrm{department}}\times \mathrm{The current cost}\;\mathrm{in}\;\mathrm{one}\;\mathrm{assistantive}\;\mathrm{department}\\ \times \frac{\mathrm{he}\;\mathrm{service}\;\mathrm{consumption}\;\mathrm{in}\;\mathrm{one}\;\mathrm{assistantive}\;\mathrm{department}\;for\;\mathrm{disease}\;\mathrm{treatement}}{\mathrm{The}\;\mathrm{service}\;\mathrm{consumption}\;\mathrm{in}\;\mathrm{one}\;\mathrm{assistantive}\;\mathrm{department}} \end{array}$
Third-level apportionment	Apportioned by the proportion of income	Department of Medical Care Design	Costs of the Department of Medical Care Design = $\begin{array}{l} \frac{\mathrm{The}\;\mathrm{income}\;\mathrm{from}\;\mathrm{new}\;\mathrm{medical}\;\mathrm{techologies}\;\mathrm{or}\;\mathrm{serivce}\;\mathrm{in}\;\mathrm{the}\;\mathrm{department}}{\mathrm{The}\;\mathrm{income}\;\mathrm{from}\;\mathrm{new}\;\mathrm{medical}\;\mathrm{techologies}\;\mathrm{or}\;\mathrm{serivce}\;\mathrm{in}\;\mathrm{the}\;\mathrm{hospital}}\times \mathrm{The current cost of deparment of medical care design}\\ \times \frac{\mathrm{The}\;\mathrm{cost}\;\mathrm{of}\;\mathrm{medical}\;\mathrm{technologies}\;\mathrm{or}\;\mathrm{service}\;\mathrm{in}\;\mathrm{this}\;\mathrm{disease}\;\mathrm{treatement}}{\mathrm{The}\;\mathrm{income}\;\mathrm{from}\;\mathrm{new}\;\mathrm{medical}\;\mathrm{techologies}\;\mathrm{or}\;\mathrm{serivce}\;\mathrm{in}\;\mathrm{the}\;\mathrm{department}} \end{array}$

Taking the data from hip replacement, acute simple appendicitis, and heart bypass surgery as examples, the full cost accounting results are shown in Tables 8–10.

**Table 8:** DRG costing results for “Hip Replacement.”

Cost category	Cost line items	Full cost	Direct costs	Indirect costs	Data sources
1. Knowledge-based human capital of medical workers	Basic quality capital	10,507	5, 668	4,839	Workload statistics report
	Job performance	77, 511	25,092	52,419	
	levels of work contribution	20, 890	9,512	11,378	
2. Drug costs	Western	26,354	26,364		List of expenses
	Proprietary Chinese medicines	15	15		List of expenses
	Chinese herbal medicine	0	0		List of expenses
3. Hygienic materials	Blood transfusion costs	0	0		List of expenses
	Oxygen usage costs	215	215		List of expenses
	Fee for image materials	2,037	2,037		Department costs, revenue
	Fees for laboratory materials	6, 480	6,480		Department costs, revenue
	Other hygiene fees	0			
	Fee-based materials	469, 941	469,941		List of expenses
	No fee for materials	2,989	2,989		Department costs
4. Depreciation of fixed assets		4,529	2,850	1,678	Department cost and workload statistical report
5. Amortization of intangible assets	Amortization of intangible assets	705		705	Department cost and workload statistical report
6. Medical risk compensation	Medical risk compensation	1,558	1,558		Department costs
Other operating costs	Other operating costs	11,347	4,114	7,233	Department costs
Total cost of disease		635,087	556,835	78,252	
Number of diseases		11			
Average full cost of disease		57,735		7, 114	

**Table 9:** DRG costing results for “acute simple appendicitis.”

Cost category	Cost line items	Full cost	Direct costs	Indirect costs	Data sources
1. Knowledge-based human capital of medical workers	Basic quality capital	400,610	251,880	148,730	Workload statistics report
	Job performance	177,670	100,750	76,920	
	Levels of work contribution	102,790	75,210	27,580	
2. Drug costs	Western	700,826	700,826		List of expenses
	Proprietary Chinese medicines	20,524	20,524		List of expenses
	Chinese herbal medicine	0	0		List of expenses
3. Hygienic materials	Blood transfusion costs	80,758	80,758		List of expenses
	Oxygen usage costs	33,500	33,500		List of expenses
	Fee for image materials	354,570	354,570		Department costs, revenue
	Fees for laboratory materials	181,560	181,560		Department costs, revenue
	Other hygiene fees	0			
	Fee-based materials	419, 980	419,980		List of expenses
	No fee for materials	121,780	121,780		Department costs
4. Depreciation of fixed assets		214,940		214,940	Department cost and workload statistical report
5. Amortization of intangible assets	Amortization of intangible assets	17,705		17,705	Department cost and workload statistical report
6. Medical risk compensation	Medical risk compensation	5,829	5,829		Department costs
Other operating costs	Other operating costs	11,347	4,114	7,233	Department costs
Total cost of disease		2,190,076	1,696,968	493,108	
Number of diseases		214			
Average full cost of disease		10,234		2,304	

**Table 10:** Cost accounting results of DRG disease “Heart bypass surgery.”

Cost category	Cost line items	Full cost	Direct costs	Indirect costs	Data sources
1. Knowledge-based human capital of medical workers	Basic quality capital	1,190,500	661,390	529,110	Workload statistics report
	Job performance	89, 860	54,340	35,520	
	levels of work contribution	30, 170	22,590	7, 580	
2. Drug costs	Western	166,755	166,755		List of expenses
	Proprietary Chinese medicines	10, 870	10, 870		List of expenses
	Chinese herbal medicine	0	0		List of expenses
3. Hygienic materials	Blood transfusion costs	95,070	95,070		List of expenses
	Oxygen usage costs	166,810	166,810		List of expenses
	Fee for image materials	120,400	120,400		Department costs, revenue
	Fees for laboratory materials	90,770	90,770		Department costs, revenue
	Other hygiene fees	0			
	Fee-based materials	1, 089, 140	1,089,140		List of expenses
	No fee for materials	16,120	16,120		Department costs
4. Depreciation of fixed assets		34,918	23,044	11,874	Department cost and workload statistical report
5. Amortization of intangible assets	Amortization of intangible assets	7,980		7,980	Department cost and workload statistical report
6. Medical risk compensation	Medical risk compensation	7,643	7,643		Department costs
Other operating costs	Other operating costs	12,712	5, 850	6,862	Department costs
Total cost of disease		3,129,718	2,530,792	598,926	
Number of diseases		75			
Average full cost of disease		41,730		7, 114	

## Discussion

In this study, considering the compensation of the value of the intellectual capital of medical workers, the cost of DRG diseases was calculated. This calculation is achieved by considering four primary indicators: technical difficulty, labor intensity, risk degree, and operation time of the disease project. In addition, factors such as the complexity of the operation steps, knowledge requirements, decision-making ability, physical exertion, concentration levels, and the likelihood of potential safety hazards to patients and occupational exposure to medical workers are taken into account. Through the refinement of eight secondary-level indicators related to service item operation time, the study calculates the knowledge capital value compensation for the disease and establishes a comprehensive cost accounting model. This endeavor addresses a longstanding issue in China’s disease cost accounting, which historically neglected the value of medical workers’ knowledge-based capital. The study has successfully redefined the positive incentive mechanism, stimulated endogenous motivation within the medical service system, instigated independent changes in medical behavior, and achieved goals of cost control and rational expenditure. Ultimately, this study provides decision-makers with valuable insights and a viable policy pathway for reforming the implementation of DRG payment systems.

## Conclusion

This study establishes a framework for compensating the knowledge capital value in cost accounting of DGR diseases, with the objective of promoting recognition of medical workers’ knowledge capital. The initiative serves a dual purpose: first, it incentivizes medical workers, fostering enthusiasm and creativity in optimizing and standardizing the diagnosis and treatment process; second, acting as a guiding mechanism for values, it addresses existing behaviors among medical workers, such as excessive prescriptions and examinations, thereby reducing disease costs in support of DRG payment reform. Subsequently, hospitals conduct disease cost accounting based on the knowledge value compensation of medical workers, thereby enhancing the transparency and authenticity of DRG pricing; furthermore, this strategy facilitates the monitoring of medical institutions’ inadequacies through DRG cost accounting.

## Data availability statement

The original contributions presented in the study are included in the article/supplementary material, further inquiries can be directed to the corresponding author.

## Author contributions

JD: Conceptualization, Data curation, Funding acquisition, Investigation, Methodology, Validation, Visualization, Writing – original draft, Writing – review & editing. FJ: Data curation, Formal analysis, Writing – review & editing. JX: Data curation, Formal analysis, Writing – review & editing. QZ: Conceptualization, Funding acquisition, Writing – review & editing.
